# Determination of the Stage of Periodontitis with 20 ng/mL Cut-Off aMMP-8 Mouth Rinse Test and Polynomial Functions in a Mobile Application

**DOI:** 10.3390/diagnostics15111411

**Published:** 2025-06-02

**Authors:** Miika Penttala, Timo Sorsa, Julie Toby Thomas, Andreas Grigoriadis, Dimitra Sakellari, Vaibhav Sahni, Shipra Gupta, Pirjo Pärnänen, Tommi Pätilä, Ismo T. Räisänen

**Affiliations:** 1 Department of Oral and Maxillofacial Diseases, Head and Neck Center, University of Helsinki and Helsinki University Hospital, 00290 Helsinki, Finland; timo.sorsa@helsinki.fi (T.S.); drthomastoby@gmail.com (J.T.T.); vaibhav.sahni@rcsed.net (V.S.); pirjo.parnanen@helsinki.fi (P.P.); ismo.raisanen@helsinki.fi (I.T.R.); 2 Division of Oral Diseases, Department of Dental Medicine, Karolinska Institutet, 171 77 Stockholm, Sweden; 3 Department of Preventive Dentistry, Periodontology and Implant Biology, Faculty of Health Sciences, Dental School, Aristotle University of Thessaloniki, 541 24 Thessaloniki, Greece; andreasgrigor@gmail.com (A.G.); dimisak@gmail.com (D.S.); 4 Dental Sector, 424 General Military Training Hospital, 564 29 Thessaloniki, Greece; 5 All India Institute of Medical Sciences (AIIMS), New Delhi 110029, India; 6 Oral Health Sciences Centre, Post Graduate Institute of Medical Education & Research, Chandigarh 160012, India; shipra1472@gmail.com; 7 Department of Pediatric Surgery, New Children’s Hospital, University of Helsinki and Helsinki University Hospital, 00290 Helsinki, Finland; tommi.patila@hus.fi

**Keywords:** periodontitis, POCT, aMMP-8, personalized medicine, logistic regression

## Abstract

**Background:** We propose a framework for determining the stage of periodontitis in a personalized medicine context, building on our previously developed model for periodontitis detection. In this study, we improved the earlier model by incorporating additional components to form a comprehensive system for identifying both the presence and stage of periodontitis. Central to the home-use application is an active-matrix metalloproteinase-8 (aMMP-8) mouth rinse test (cut-off: 20 ng/mL), integrated with software delivered via a mobile application. **Methods:** First, using all the data, we modeled a single polynomial function to distinguish healthy and stage I periodontitis patients from stage II and III patients. Second, we used an already published periodontitis detection function to separate stage I patients from healthy patients. Third, one more function was created that divided stage II and III patients from each other. All functions were modeled by multiple logistic regression analysis from the patient data, which consisted of 149 adult patients visiting dental offices in Thessaloniki, Greece. **Results:** The complete model demonstrated a sensitivity of 95.8% (95% CI: 92.1–99.4%) and a specificity of 71.0% (95% CI: 55.0–86.9%) for detecting periodontitis. Among those identified with periodontitis, the correct stage was determined in 61.1% of cases, with stage-specific accuracies of 64.3% for stage I, 60.5% for stage II, and 60.9% for stage III. All testing was performed on patient data with which the complete model was formed. **Conclusions:** The results of this study showed that with sufficient data and using multiple logistic regression analysis, a model can be created to simultaneously identify the presence and stage of periodontitis. Overall, in the complete model generated, a mouth rinse aMMP-8 test result with a cut-off value of 20 ng/mL, Visible Plaque Index (VPI) and information of patient’s teeth number present were found to be important factors to determine the stage of periodontitis in a personalized medicine manner for everyone to use.

## 1. Introduction

Periodontitis is a common chronic inflammatory disease characterized by destruction of the supporting structures of the teeth. The disease and its stages are associated with many conditions, e.g., diabetes and cardiovascular diseases [1,2,3,4]. According to data from the 2010s, it is estimated that half of dentate adults worldwide have some category of periodontitis [5,6]. Severe (stage III) periodontitis affects approximately 10% of the adult population and is characterized by advanced symptoms, including tooth loss caused by the disease [7,8,9]. Early diagnosis is crucial, as the clinical progression of periodontitis can be halted or mitigated. This can be achieved by clinically successful anti-infective periodontal treatment, i.e., scaling and root planing (SRP) [4,10,11,12]. In addition, SRP treatment at chronic periodontitis can be supported and maintained by medicine use, e.g., low-dose-doxycycline anti-aMMP-8 adjunctive medication [13]. Overall, the treatment of periodontitis at any stage requires a multifaceted approach. These include, i.a., improving the patient’s self-care of their teeth, reducing risk factors related to tobacco smoking or diabetes control, and, for example, optimizing periodontitis treatment through motivational interviewing [4,14,15].

In this article, we propose a framework for creating a statistical model that detects the stage of periodontitis. The framework and idea are built on the periodontitis-revealing method introduced in the article *Periodontitis Home Screening with Mouth Rinse Cut-Off 20 Ng/mL aMMP-8 Test and Mobile Application*, published in *Diagnostics* by Penttala et al. (2025) [16]. In this study, we created more components to the model already presented, which allowed the model to also determine the stage of periodontitis. The method is non-invasive and cost-effective, and it is easy to use, for example, at home. It will empower patients to seek early dental intervention, ideally at stage I periodontitis.

In modeling the stage of periodontitis, we used results from Greece of a previous study by Sorsa et al. [17]. According to this study, an active-matrix metalloproteinase-8 (aMMP-8) mouth rinse test can be used as a biomarker to identify the stage of periodontitis in clinical practice in just five minutes. In addition, the aMMP-8 point-of-care test (POCT) with Oralyzer (i.e., aMMP-8 digital reader) has recently been repeatedly demonstrated to be the most precise biomarker in discriminating periodontal health and disease [18,19,20]. The aim of this study is to model polynomial functions that can be used to determine the stage of periodontitis using variables, e.g., aMMP-8 mouth rinse test result (cut-off value: 20 ng/mL), age and tobacco-smoking status.

## 2. Methods and Materials

### 2.1. Framework and Hypothesis

We propose a framework for creating a model for determining the stage of periodontitis. In short, the idea of the complete model is that, first, a polynomial function created in this study distinguishes healthy and stage I periodontitis patients from stage II and III patients. If the patient falls into the first category, the already published periodontitis detection function separates healthy patients from stage I patients [16]. If the patient has stage II or III periodontitis, another function created in this study reveals the stage. The framework includes multiple logistic regression analyses to determine which specific variables define the stage of periodontitis with adequate accuracy.

**Figure 1:**
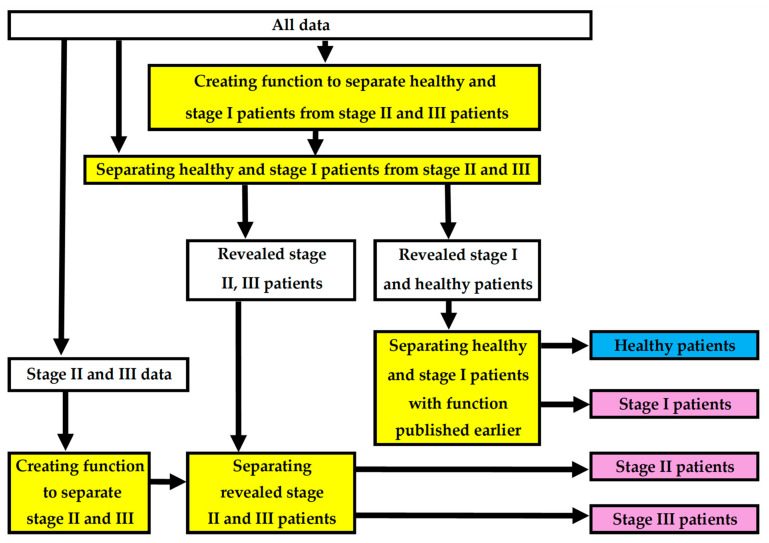
Flowchart of the framework creating and testing the complete model to determine the stage of periodontitis.

In this study, the modeling of patient’s periodontitis stage was based on three statements: (i) the aMMP-8 chairside/point-of-care (PoC) oral fluid tests identify active periodontal tissue destruction; (ii) periodontitis is a biofilm-associated inflammatory disease of the periodontium; (iii) periodontitis is a major cause of tooth loss in the world [9,21,22]. This study also examined other variable candidates to determine the stage of periodontitis, such as tobacco-smoking or diabetes status, etc. The approach of this research is entirely statistical. Patients revealed to have periodontitis at any stage are referred to a dentist for follow-up. Figure 1 shows the flow diagram of the framework used in this study.

### 2.2. Periodontitis Classification

All patients in the dataset were classified according to the 2018 classification of periodontal diseases [8,23]. In logistic regression analysis, later in this study, different stages of periodontitis were examined as binary values. For example, to distinguish stage II periodontitis patients from stage III patients, stage II patients were recorded as zero and stage III patients as one.

### 2.3. Staging of Periodontitis

Periodontitis staging is determined using Clinical Attachment Loss (CAL) or radiographic bone loss (RBL). Also, information on tooth loss that can be attributed primarily to periodontitis and the periodontal probing depth (PPD) are used in the staging of periodontitis [8,23].

Stage I periodontitis is the borderland between gingivitis and periodontitis and represents the early stages of attachment loss. A patient is a periodontitis case (i.e., at least stage I patient) in the context of clinical care if: (i) Interdental CAL is detectable at ≥2 non-adjacent teeth, or (ii) Buccal or oral CAL ≥ 3 mm with pocketing > 3 mm is detectable at ≥2 teeth and (iii) the observed CAL cannot be ascribed to non-periodontal causes such as (1) gingival recession of traumatic origin; (2) dental caries extending in the cervical area of the tooth; (3) the presence of CAL on the distal aspect of a second molar and associated with malposition or extraction of a third molar; (4) an endodontic lesion draining through the marginal periodontium; and (5) the occurrence of a vertical root fracture [8,23].

Stage II represents established periodontitis, in which a carefully performed clinical periodontal examination identifies the characteristic damages that periodontitis has caused to tooth support (e.g., CAL 3 to 4 mm). At stage III, periodontitis has produced significant damage to the attachment apparatus (e.g., CAL ≥ 5 mm) and, in the absence of advanced treatment, tooth loss may occur [8,23]. No stage IV patients were found in the research data and therefore they are excluded from this study, but it is worth mentioning that at a more advanced stage IV condition, periodontitis causes considerable damage to the periodontal support and may cause significant tooth loss, and this translates to loss of masticatory function [8,23].

### 2.4. aMMP-8 Point-of-Care Testing (POCT)

The patients’ mouth rinse aMMP-8 levels were used to determine the stages of periodontitis based on the findings that stage I patients are associated with levels < 20 ng/mL and stage III patients with levels ≥ 20 ng/mL [17,24]. The aMMP-8 POCT can measure and assess active and progressive collagenolytic periodontal and peri-implant attachment loss due to aMMP-8 within five minutes by a non-invasive procedure, i.e., without invasive tissue examination and bacteremia [17,18,20,25,26,27,28,29,30,31,32,33,34,35,36,37].

The levels of aMMP-8 test were analyzed quantitatively by the chairside/PoCPerioSafe^®^ (Medix Biochemica Ltd., Espoo, Finland) immunotest accompanied by the digital reader ORALyzer^®^ (Dentognostics GmbH, Solingen, Germany) according to the manufacturer’s instructions [30]. The aMMP-8 test was conducted by a trained and calibrated (AG) researcher on this study’s patients. The test is performed as follows: (1) 30-second pre-rinse with potable tap water; (2) 1 min wait after pre-rinse; (3) 5 mL of test solution (oral fluid sample) is collected after 30 s of rinsing; (4) three drops of the sample solution are transferred to the test system; (5) the result can be read on the reader within 5–6 min; (6) the test result is positive if the aMMP-8 concentration is ≥20 ng/mL [16,38,39].

The aMMP-8 chair-side oral fluid lateral flow immunotest was independently and repeatedly confirmed by many parties [18,25,26,27,28,29,30]. These aMMP-8 tests with a cut-off of 20 ng/mL were successfully clinically and independently validated in Finland, Nigeria, Germany, Holland, Mali, Türkiye, India, Italy, Chile, Sweden, and the USA [18,19,20,33,36]. The PerioSafe^®^ LF aMMP-8-POCT kits(Medix Biochemica Ltd., Espoo, Finland) are efficient and handy tools for the improvement of diagnostic and prognostic accuracy for periodontal or peri-implant diseases, and are commercially available and approved technologies by the FDA/USA and the EU [18].

### 2.5. Dental Plaque

The amount of plaque on patients’ teeth was estimated using the Visible Plaque Index (VPI) [40]. The method describes the amount of visible plaque on the teeth. The patients’ VPI was calculated as follows: “The method estimates the presence of biofilm on tooth surfaces by adding up the presence of dental plaque on individual tooth surfaces and dividing it by the number of all surfaces”. VPI was chosen as the plaque assessment method for this study because it is within the reach of every person. As the statistical estimation method develops, this can be ensured with good operating instructions.

By using the VPI, we were able to investigate the link between biofilm on tooth surface and the stage of periodontitis. It is understood that the primary etiological agent for the initiation and progression of periodontal disease is the dental plaque biofilm [41]. To maximize the full potential of VPI in the statistical model, we also investigated VPI%, along with other factors, to obtain a more complete explanatory variable for the model being created. These factors included information such as the patients’ annual dental visits or the number of teeth present. This approach involved investigating whether a VPI cut-off value, e.g., VPI% < 50% and, e.g., data on annual dental visits or missing teeth, could together produce more accuracy to the determination of stage of periodontitis.

### 2.6. Missing Teeth

Periodontitis is the major cause of tooth loss in the adult population worldwide and the number of lost teeth because of periodontitis is a parameter determining the stages of periodontitis [8,9,23]. Therefore, we wanted to investigate whether simple information about the number of teeth a patient has would improve the statistical model. The assumption of complete dentition was met if the patient had 28 teeth excluding wisdom teeth.

### 2.7. Sample Data and Parameters

A total of 149 Greek adult patients attending a Periodontology University Clinic and General Army Hospital in Thessaloniki were enrolled in this study. Oral health status was recorded for all patients by one calibrated examiner (A.G.), as previously described in detail [16,38]. Clinical periodontal and oral health parameters were assessed for six surfaces of each tooth using an automated probe (Florida probe, Florida Probe, FloridaProbeCorporation, Gainesville, FL, USA). Third molars were excluded [16,38].

The participants signed an informed consent form, and this study was conducted according to the protocol outlined by the Research Committee, Aristotle University of Thessaloniki, Greece, and approved by the Ethical Committee of the School of Dentistry (protocol No. 64, 12 June 2018). All procedures performed in the present study involving human participants were in accordance with the ethical standards of the institutional and/or national research committee and with the 1964 Helsinki Declaration and its later amendments, or comparable ethical standards [38,39].

Inclusion criteria: age ≥ 18 years, presence of ≥20 teeth, patient physical status 1 or 2 in the American Society of Anesthesiology classes, score ≥ 9 on the questionnaire for patients at high risk of developing diabetes (Centers for Disease Control and Prevention (CDC, United States)). Exclusion criteria: presence of diabetes or immunomodulatory diseases, medication that affects glucose balance, periodontal therapy for the last 6 months, women in pregnancy or lactation.

Patient data were recorded for general characteristics, taking into account potential risk factors for periodontitis., e.g., gender, age, tobacco-smoking status (yes/no) and waist circumference (Table 1) [16,38]. This cross-sectional observational study was conducted in 2017–2018. There were no issues with missing data, and we followed the STROBE statement in reporting this study.

### 2.8. Logistic Regression and Assumptions

All polynomial functions developed in this study were created using multiple logistic regression with a backward stepwise variable selection technique [42]. Multicollinearity was checked with variance inflation factor (VIF), and it was calculated for explanatory variables by running a linear regression of one explanatory variable on the other explanatory variables and then obtaining the R^2^ result from that regression. The VIF was calculated by formula (1 − R^2^)^−1^ [43]. Autocorrelation was investigated by creating a scatterplot of residuals versus time, where patient codes in chronological order represented time. The linearity assumption of logistic regression analysis was also checked with a scatterplot if the variables of the generated functions included continuous variables.

### 2.9. Sample Size

It was decided that all polynomial functions generated in this study should include at least 100 patients. This was supported by Long’s statement that the sample size for multiple logistic regressions should not be lower than 100 [44]. It was also important that the number of events per variable (EPV) was at least 10 for all functions to be developed. According to Peduzzi et al., having fewer than 10 events per variable (EPV) can lead to major problems [45]. (Number of events = lower total number of possible outcomes). Therefore, we set the multiple logistic regression criterion that the EPV must be ≥10.

### 2.10. Statistical Analysis

To determine the performance of the polynomial functions, sensitivity, specificity, accuracy and F1 score analyses were performed. Also, the phi coefficient φ was calculated for categorical data and the Chi-square goodness of fit test was used to evaluate the developed functions. The receiver operating characteristic (ROC) curve was used to assess the overall diagnostic performance of the polynomial functions. To maximize sensitivity and specificity, Youden’s index was applied. The maximized Youden’s index was agreed to show the optimal cut-off value of the functions, e.g., to distinguish stage II periodontitis patients from stage III patients. The maximized Youden’s index was calculated with the following formula: sensitivity + specificity − 1, where sensitivity and specificity can have values ≥ 0 and ≤1 [46]. The values of the ROC curve, which produced the maximal result of the formula mentioned, were chosen for sensitivity and specificity values. The area under the ROC curve (AUC) was also calculated. In addition, the analysis included a particularly careful examination of samples with a Cook’s distance > 0.5 or a standardized distance greater than ±3.0. The data were analyzed using IBM SPSS Statistics (Version 29) software. The significance level for all variables in the generated polynomial functions was set at *p* ≤ 0.05.

## 3. Results

### 3.1. Created New Two Functions

Two new polynomial functions were created in this study: PERIOSTAGE I and PERIOSTAGE II/III. The former separates healthy and stage I patients from stage II and III patients, the latter distinguishes stage II and III patients from each other. Both functions passed the chi-square goodness-of-fit test and the phi coefficient φ results for categorical data, F1 score and AUC values were deemed sufficient and acceptable. The PERIOSTAGE I function exhibited a sensitivity of 79%, specificity of 76%, and accuracy of 78%. The PERIOSTAGE II/III function had a sensitivity of 65%, specificity of 80%, and accuracy of 77%. Testing of both functions was performed using the patient data from which they were formed.

- PERIOSTAGE I: The phi coefficient φ = 0.52; F1 score 0.83; chi-square test χ^2^ (1, N = 149) = 3.46, *p* = 0.06; AUC = 0.842 (95% CI = 0.774–0.910), sensitivity 82/104 = 0.79; specificity 34/45 = 0.76; accuracy 116/149 = 0.78; Youden’s index = 0.5441, cut-off 0.598.
- PERIOSTAGE II/III: The phi coefficient φ = 0.41; F1 score 0.56; chi-square test χ^2^ (1, N = 104) = 2.94, *p* = 0.09, AUC = 0.779 (95% CI = 0.680–0.878), sensitivity 15/23 = 0.65; specificity 65/81 = 0.80; accuracy 80/104 = 0.77; Youden’s index = 0.4545, cut-off 0.326).

**Figure 2:**
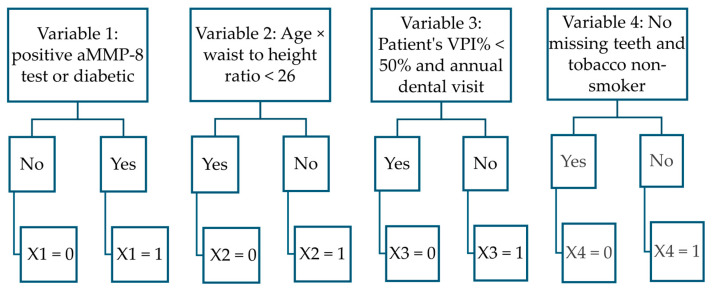
Flowchart illustrates the variables in separating healthy and stage I patients from stage II and III patients.

This study identified key factors in the polynomial function derived from the Greek dataset that most accurately distinguish healthy individuals and stage I periodontitis patients from those with stage II and III. These factors include aMMP-8 concentration < 20 ng/mL in mouth rinse, age, waist-to-height ratio, Visible Plaque Index < 50%, presence of a full set of teeth, annual dental visits, and diabetes and tobacco-smoking status (Figure 2).

**Figure 3:**
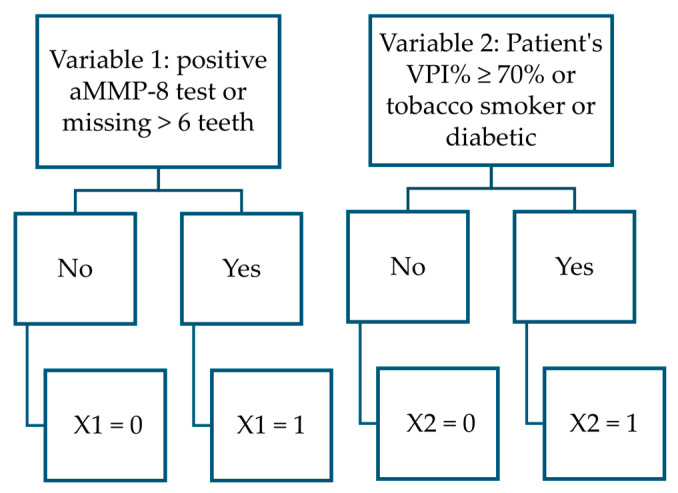
Flowchart illustrates variables in differentiating stage II and III periodontitis patients.

The most accurate variables in distinguishing stage II from stage III periodontitis in this dataset were the aMMP-8 test result (≥20 ng/mL), diabetes and tobacco-smoking status, VPI (≥70%), and the presence of more than six missing teeth (Figure 3). The coefficient output data from the logistic regression modeling are presented in Table 2.

The PERIOSTAGE I function had a sample size of 149, with 45 events (i.e., the lower total number of possible outcomes) and four variables were used in modeling, resulting in events per variable (EPV) of 10.3 (i.e., >10). The PERIOSTAGE II/III function had a sample size of 104, with an EPV of 23 divided by 2. The result of 23/2 is 11.5 (i.e., >10). Furthermore, as both functions were modeled using data from over 100 patients, the sample size for the polynomial functions presented in this study was considered sufficient based on studies of Long and Peduzzi et al. [44,45].

The linearity assumption in logistic regression was satisfied for both functions, as all variables were binary. An examination of the scatterplots between residuals and time (with patient codes in chronological order representing time) revealed no autocorrelation in either function. Multicollinearity was assessed using the variance inflation factor (VIF) values, which indicated no problematic correlations among the independent variables (the VIF values ranged from 1.064 to 1.163 for PERIOSTAGE I and were 1.000 for both explanatory variables in PERIOSTAGE II/III). No sample had a Cook’s distance value above 0.5 or a standardized residual exceeding three. All performance calculations for the two functions created in this study were found to meet the required level of approval. This supported the conclusion that the developed polynomial functions represented sufficient performance in this study.

### 3.2. Forming of the Complete Model

**Figure 4:**
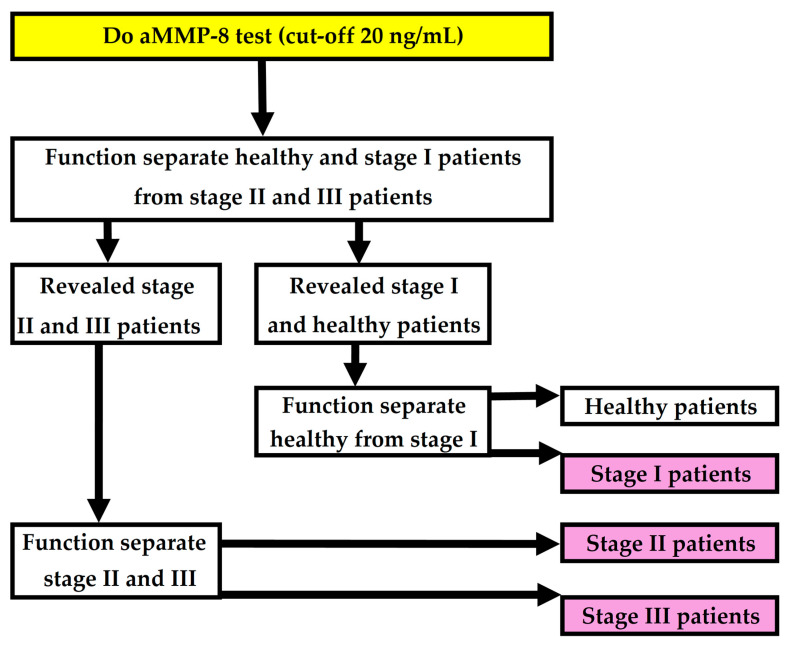
Flowchart for revealing periodontitis and the stage of the condition.

The complete periodontitis detection model presented in this study consists of three polynomial functions. The first function distinguishes healthy and stage I periodontitis patients from stage II and III patients. The second function, which is based on a previously published periodontitis detection model [16], separates stage I patients from healthy patients. The third function further divides stage II and stage III patients. Together, these three polynomial functions form the complete model (Figure 4). The polynomial function used to separate healthy and stage I periodontitis patients from stage II and III patients is as follows:

$$PERIOSTAGE\;I\;=\frac{1}{1+e^{-\left(1.997\times X1+1.615\times X2+1.653\times X3+1.180\times X4-2.649\right)}}$$

In which:

PERIOSTAGE I = probability of patient statistically found healthy or having stage I periodontitis [No.] (if the result ≥ 0.598, the patient statistically has stage II or III; otherwise, the patient is statistically considered healthy or has stage I periodontitis).

X1 = a positive aMMP-8 POCT outcome or a diabetic [No.] (yes = 1, a negative aMMP-8 POCT outcome and non-diabetic patient = 0).

X2 = age × waist-to-height ratio < 26 (with two decimal places, i.e., 25.99 < 26.) [No.] (yes = 0, no = 1, patient boundary conditions are: waist-to-height ratio 13.50–56.42, 25 yrs. ≤ age ≤ 78 yrs., WC 60–152 cm, height 150–193 cm).

X3 = Visible Plaque Index (VPI) score < 50% (with two decimal places, i.e., 49.99% < 50%.) and visits annually dental clinics [No.] (yes = 0, no = 1).

X4 = the patient has a full set of teeth and is a tobacco non-smoker [No.] (yes = 0, no = 1, full set of teeth = number of teeth equals 28, wisdom teeth are excluded from the count)

The polynomial function to determine whether a patient is healthy or have stage I periodontitis by Penttala et al. (2025) [16] is as follows:

$$PERIORISK\;=\;\frac{1}{1+e^{-\left(3.392\times X1+0.002\times X2+1.858\times X3-9.151\right)}}$$

In which:

PERIORISK = probability of patient not having periodontitis [No] (if the result < 0.55, the patient is statistically considered not to have periodontitis, otherwise = the patient statistically has periodontitis)

X1 = a positive aMMP-8 POCT outcome or a tobacco smoker [No.] (yes = 1, a negative aMMP-8 POCT outcome and tobacco non-smoker = 0).

X2 = age x waist circumference [yrs. x cm] (possible input 2200–8772, patient boundary conditions are: 25 yrs. ≤ age ≤ 78 yrs., WC 60–152 cm).

X3 = patient is diabetic or patient’s parent is diabetic [No.] (yes = 1, otherwise = 0) [16].

Further on, if the patient was not determined to be included into the healthy or periodontitis stage I categories, the patient data are to be run through one more function to separate the remaining stage II and stage III patients from each other.

The polynomial function created to separate periodontitis stage II and III patients is as follows:

$$PERIOSTAGE\;II/III\;=\;\frac{1}{1+e^{-\left(1.719\times X1+1.988\times X2-3.706\right)}}$$

In which:

PERIOSTAGE II/III = probability of periodontitis patient having stage III condition [No.] (if the result ≥ 0.326, the patient statistically has stage III; otherwise, the patient is statistically considered to have stage II).

X1 = a positive aMMP-8 POCT outcome or the number of missing teeth is > 6 [No.] (yes = 1, a negative aMMP-8 POCT outcome and less than 6 teeth are missing = 0, wisdom teeth are excluded from the count).

X2 = Visible Plaque Index (VPI) score ≥ 70% (with two decimal places, i.e., 69.99% < 70%.) or is tobacco smoker or diabetic [No.] (yes = 1, no = 0).

### 3.3. Performance of the Complete Model

The sensitivity of the complete model to detect periodontitis was found to be 95.8% (95% CI = 92.1–99.4%) and the specificity was 71.0% (95% CI = 55.0–86.9%); (Se 113/118 = 0.958; Sp 22/31 = 0.710). Among those identified with periodontitis, the correct stage was determined in 61.1% of cases (95% CI = 52.1–70.1%); (Acc. 69/113 = 0.611), with stage-specific accuracies of 64.3% for stage I, 60.5% for stage II, and 60.9% for stage III; (stage I: 9/14 = 0.643; stage II = 46/76 = 0.605; stage III 14/23 = 0.609). In addition, 95% confidence intervals were determined for stages I, II and III (95% CI = 39.2–89.4%), (95% CI = 49.5–71.5%), (95% CI = 40.9–80.8%), respectively. Testing of the complete model was performed using the patient data from which it was formed. All performance calculations for the complete model created in this study were found to meet the required level of approval. This supported the conclusion that the generated complete model represented sufficient performance in this study.

## 4. Discussion

The model developed in this study provides a tool for personalized medicine in determining the stages of periodontitis, allowing patients to seek early dental intervention, ideally in stage I of periodontitis. Central to this approach is the aMMP-8 mouth rinse test (cut-off value: 20 ng/mL), integrated with the software described, which can be deployed via a mobile application. This system identifies periodontitis and its stages using accessible statistical factors. In the complete model created, the result of the aMMP-8 test (cut-off value: 20 ng/mL), the Visible Plaque Index, and the number of teeth present were found to be statistically important variables in determining the stages of periodontitis.

To reveal the correct stage, it was essential to perform the aMMP-8 test (cut-off value: 20 ng/mL). It is noteworthy that, already in 2020, Sorsa et al. proposed the testing of aMMP-8 PoC/chair mouth rinse to be incorporated into the new periodontal disease classification system by Tonetti et al. [8,17]. We used this proposal and hypothesized that since stage I periodontitis patients are associated with aMMP-8 levels below 20 ng/mL, we could use this information to statistically distinguish between stage I and rest of the periodontitis patients [17,24]. Furthermore, it can be concluded that since the concentration of aMMP-8, ≥20 ng/mL, is associated with stage III periodontitis, we could use a cut-off value of 20 ng/mL to statistically separate stage II and III patients as well [17,24]. In this way, the cut-off value of 20 ng/mL could also be used for staging and not only for separating healthy patients from periodontitis patients.

In our study, we also wanted to test the ability of higher cut-offs, such as 40 or 60 ng/mL, to differentiate between stage II and stage III patients. However, their correlation values were still of the same order of magnitude as the 20 ng/mL cut-off. The correlation values for all of the above cut-offs represented moderate correlation values. (The phi coefficient φ for categorical data for separating stage II and stage III patients from each other for cut-off values of 20, 40 and 60 ng/mL were 0.30, 0.35 and 0.36, respectively.) It can therefore be concluded that while higher cut-off values might streamline detection of severe cases to some extent, they risk underdiagnosing milder or early-stage disease, which is critical for preventive care.

The VPI% value was analyzed with other factors such as annual dental visits and the number of teeth present. The combined explanatory variable was found to improve the accuracy of the model in creation. This approach revealed that VPI% < 50%, combined with annual dental visit data, effectively distinguished healthy individuals and stage I periodontitis patients from those with stage II or III periodontitis (Figure 2). Conversely, VPI% ≥ 70% was a useful factor in differentiating stage II from stage III periodontitis (Figure 3). The association was supported by the theory that the primary etiological agent for the initiation and progression of periodontal disease is the dental plaque biofilm [41]. Dental plaque is considered a structurally and functionally organized biofilm [47]. However, the data from this study only included information on the amount of plaque. No attempt could be made to model how, e.g., microbial specificity would affect the results of determining the stages of periodontitis [48]. This would be one possible research scenario in future modeling with more accurate sample data.

The VPI was included in this study because it is in reach for every person and as the staging method develops, dental plaque examination can be ensured with good operating instructions. An interesting additional observation of this study, which requires further investigation, was that it appears possible to assess the Visible Plaque Index with sufficient accuracy by investigating maxillary incisors alone. One example is a correlation value obtained from research data involving a group of patients with stage II or III periodontitis. The VPI and the amount of visible plaque in the maxillary incisors were found to be associated with a strong positive correlation, r = 0.82, which was calculated using Pearson’s correlation coefficient. If plaque was detected on any part of the labial surface of a maxillary incisor, the plaque score for that tooth was recorded as 1; if visible plaque was not observed, the score was 0. For example, if a patient had four maxillary incisors, and one tooth exhibited visible plaque, the value was calculated to be 0.25. We observed that the assessment of visible plaque on teeth surface could possibly be significantly facilitated by this method. A simple estimate of the amount of plaque on certain teeth and its correlation with the VPI appears to be strong, but this matter needs to be investigated using diverse datasets.

The patient’s annual dental visits proved to be an interesting factor in determining the stage of periodontitis and were used in modeling in a special composite variable (Function PERIOSTAGE I, variable X3 = the patient’s number of plaque-free teeth is <50% and visits annually dental clinics). However, e.g., dental fear could be an explanatory factor that enhances the model’s accuracy combined with annual dental visit information. People with higher dental fear have been found to visit the dentist less often [49]. On the other hand, the severity of periodontitis has been found to have an association on a short time period between oral health examinations [50]. This paradox could be explained by the knowledge that severe periodontitis affects approximately 10% of the dentate population. Thus, annual dental visits could work as a statistical parameter when screening healthy and stage I patients, who represent the majority of the dentate population [7,9]. Annual dental visits as a variable is worth studying with great interest, as it is easy to ask patients about and may prove to be a very important variable in the further development of modeling.

If the patient was determined to be healthy or to have stage I periodontitis, we wanted to separate these patients from each other. For this task, we used a function previously published by Penttala et al. 2025 [16] (Figure 4). The aforementioned function was created to reveal whether a patient has periodontitis. And since this function was exactly what we needed to distinguish stage I patients from healthy ones, it was reasonable to use an already published function for this task.

However, if a patient was determined to belong to stage II or III, it was necessary to create one more function that could differentiate these patients from each other. By succeeding in this, as written in the next chapter, we formed a model that revealed the three stages of periodontitis, which was also the goal of this article. In this study, we also explored the possibility of first revealing, e.g., stage III periodontitis patients and then continuing to differentiate healthy, stage I and stage II periodontitis patients, but this was more or less only one possibility. It is worth mentioning that the modeling process involved extensive multiple logistic regression analysis, examining numerous combinations of variables using various factors to ensure the most accurate results.

As a crucial factor in identifying stage III patients from stage II, it was found that if a stage III patient statistically met the criterion mentioned earlier in this discussion, an aMMP-8 test result ≥ 20 ng/mL, the needed accuracy was obtained in the model. In addition, to distinguish stage III patients from stage II patients in this dataset, it was essential to examine the number of teeth present. The knowledge that a patient was missing more than six teeth provided sufficient accuracy in the model for this dataset. The number of teeth present should not be confused with the criteria for the stage of periodontitis, where stage II patients do not have missing teeth due to periodontitis. The reason for this is that the method presented in this study is indeed purely statistical. However, it is worth mentioning that periodontitis is generally the most common cause of tooth loss in the adult population worldwide, and this, in turn, explains the association between the number of teeth present and the stage of periodontitis [8,9,23]. Number of teeth present was also a reasonable variable in terms of the usability of the model, as counting teeth does not require the expertise of a dentist. Other factors which were included into the function separating stage II and III were the above-mentioned VPI% ≥ 70%, diabetes status and tobacco-smoking status.

In the sample data of this study, the tobacco-smoking status data (yes/no) fit the staging model. Furthermore, the amount of smoking, not just the smoking status, has been found to be associated with the severity of periodontitis [51,52,53,54]. In the future, when developing the method presented in this study, smoking in all its forms should be studied more closely and comprehensively with complete data.

Information about the patient’s diabetes status was one of the factors in the model and its effect on the stage of periodontitis should also be investigated in detail. A clear connection has been found between the degree of hyperglycaemia and the severity of periodontitis [1]. This study was conducted in the Thessaloniki region, where approximately 10% of the adult population has diabetes [55]. In the data of this study, the prevalence of diabetes was determined by the HbA1c blood test to be 4.7% [16]. As the statistical method develops and the amount and diversity of patient data increases, the accuracy of the model can increase to a higher level. The proportion of undiagnosed diabetes is estimated to be 1.5% in adults in Greece and, for example, the United States [55,56].

Interestingly, diabetes and periodontitis are both diseases associated with aging [57,58,59]. In addition to the mentioned connection between the degree of hyperglycemia and the severity of periodontitis, it has been shown that the severity of periodontitis increases with age [1,7,60]. However, in the dataset of this study, diabetes status as a variable performed better than age in determining the stage of periodontitis. Modeling determined that diabetes status was used in all three functions in this study. Age was also used to determine the stage of periodontitis but not directly, and it was more or less used to reveal healthy patients. Regression analysis found that age multiplied by waist-to-height ratio was a valuable explanatory variable separating healthy and stage I periodontitis patients from stage II and III patients. On the other hand, age multiplied by waist circumference was used as a variable in a function that revealed stage I patients from healthy ones, as mentioned earlier in this discussion section (Figure 4) [16]. Furthermore, it is interesting that the waist circumference index (WWI) has been observed to have a positive correlation with periodontitis with a particularly pronounced impact on moderate periodontitis [61] (the WWI is calculated as waist circumference in centimeters divided by the square root of weight in kilograms and then rounded to two decimal places [62]). In this study, the WWI was not used in modeling because the waist circumference and waist-to-height ratio were found optimal for modeling.

There are numerous other potential variables to determine the stage of periodontitis. For example, in a systematic review, a positive linear association was observed, suggesting that the more severe periodontitis is, the higher the likelihood of having hypertension [63]. The use of medications or, for example, gender differences in potential risk profiles regarding the severity of periodontitis would also be examples of topics worth exploring [58,64]. As the understanding of associations and causes of periodontitis increases, many novel variables can be found to increase the accuracy of the method presented.

It was agreed that in the multiple logistic regression modeling of this study, the number of events per variable (EPV) should be at least 10 (number of events = lower total number of possible outcomes [45]). This set limitations on the number of independent variables that could be selected in modeling. For example, the determination of stages II and III had to be modeled with only two variables in this dataset. The lower total number of possible outcomes in the stage III group was relatively small, 23, and according to Peduzzi et al., modeling with more than two variables is not recommended in the function created for this modeling scenario [45].

Taking into account the above-mentioned limit, the use of small numbers of variables, and particularly the fact that only two variables could be utilized to differentiate between stage II and III patients, the periodontal phase of the patients was assessed in this study at least at a sufficient level. Among those identified with periodontitis, the correct stage was determined in 61.1% of cases, with stage-specific accuracies of 64.3% for stage I, 60.5% for stage II, and 60.9% for stage III. As the sample size increases and more explanatory variables can be used, it can be expected that the results will rise to a higher level. It should be noted that the aforementioned limitation did not prevent the presentation and introduction of this method and proposal as a new way to determine the stage of periodontitis, but it should be taken into consideration in the examination of the results.

In future development of the method, it would be necessary to have at least 30 patients in each periodontitis stage category, preferably 50 patients, which would reduce the possibility of, e.g., overfitting. This would allow for the use of possibly three or more variables. In this scenario of three stages, there would be a total of 3 times 50 patients, or a total of 150 periodontitis patients. Since the prevalence of periodontitis is approximately 50% of the world’s population, and referring to this information, healthy subjects would increase the total number of subjects in the study to 300 [5,6]. For cross-validation, a sample size of 500 patients would be practical, and 300 patients could be used for modeling (i.e., training) and 200 patients for testing the model.

It should be noted that the model created in this study is not universal. It is a model that statistically estimates the stage of periodontitis, created from the data of this study. If there is another population, the calculations have to be performed in a similar manner and the results can be different. Every population should have its own model, and its factors would probably vary between different populations, but the basic variables should be the same as those we could predict. One can conclude that, at least in theory, the variables presented in this study would be broadly applicable in the future development of the method introduced. The aMMP-8 enzyme and dental plaque on the teeth surface, both utilized in our staging model, have been found to be associated with periodontitis and its stages in many different populations [17,24,41,65]. Also, for example, the number of teeth present is linked with the stages of periodontitis, as periodontitis is the major cause of tooth loss in the adult population worldwide [8,9,23]. Tobacco smoking, diabetes, and age are other essential examples of variables in this study associated with the stages of periodontitis in many populations [1,7,51,52,53,54,60].

The method presented in this article has the potential to improve the accuracy of diagnosing the stage of periodontitis, especially when used in connection with existing methods. It has been suggested that current diagnostic challenges include errors due to, e.g., a lack of experienced dentists or limited time for radiograph analysis. Furthermore, one can predict that integrating AI innovations in periodontitis diagnosis enhances diagnostic accuracy and efficiency, providing a robust alternative to conventional methods. These technologies offer quicker, less labor-intensive, and more precise alternatives to classical approaches [66]. However, the integration of AI in dentistry should be seen as a complementary tool that enhances clinical practice rather than replacing human experts [67].

In the future, using the statistical method presented in this study, the stage of periodontitis may be confirmed in some patient cases without X-ray imaging. This could potentially reduce the number of patients exposed to X-rays. However, the method is intended for people without specialized dental knowledge. This suggests that its impact in the health sector would be to refer patients to dental clinics and thus be a new addition to the treatment of periodontitis. As the method develops, new parameters may allow the research method to be applied directly to dental offices and the accuracy of the models would potentially be increased.

To fully harness the potential of statistical models in determining periodontitis and its stages, larger datasets and more comprehensive explanatory variables are needed. Above all, it is important that all generated estimation models are externally validated before they can be accepted for use in a wider population [68]. As the current model was only tested on the dataset from which it was derived, real-world validation is essential to assess its reliability and generalizability. Regulatory testing is also a matter that will have to be met by all possible standards. This way, it will be ensured that the software application meets a legal set of standards and requirements.

It is essential to highlight that the purpose of this research is to introduce a screening method for home use, which is not intended to replace current diagnostic methods. Conventional approaches, e.g., periodontal probing and radiographic analysis, cannot be replaced by the statistical estimation models that this study offers. The presented method enables individuals to estimate their statistical probability of having periodontitis and estimate its stage proactively. Additionally, it can assist healthcare providers in identifying at-risk patients and recognizing, e.g., systemic conditions associated with periodontitis [1,2,3,4].

## 5. Conclusions

To our knowledge, this is the first home-use model developed to estimate the stage of periodontitis using a mouth rinse aMMP-8 test. Utilizing a test result (cut-off value: 20 ng/mL) with multiple logistic regression analysis offers a non-invasive, cost-effective, and accessible periodontitis staging method for everyone to use. This study demonstrates that with sufficient data, a complete model can be created to detect both the presence and severity of periodontitis. We propose broader adoption of this method in periodontology as it holds promise for evolving into a widely usable tool in personalized medicine.

## Figures and Tables

**Table 1:** Comparison of the general characteristics in the dataset. The data are presented as mean ± SD for continuous variables and as a number for the categorical variables. aMMP-8, active-matrix metalloproteinase-8; CAL, clinical attachment level; PPD, periodontal probing depth; VPI, Visible Plaque Index; BOP, bleeding on probing.

Patient’s Data	Periodontal Status			
	No Evidence of Periodontitis (*n* = 31)	Stage I (*n* = 14)	Stage II (*n* = 81)	Stage III (*n* = 23)
**Gender**				
Female	11	13	39	12
Male	20	1	42	11
**Smoking status**				
Tobacco smoker	3	7	24	10
Non-smoker	28	7	57	13
**Diabetic status**				
Diabetic	0	0	3	4
Non-diabetic	31	14	78	19
**Annual dental visit**				
Yes	19	13	45	13
No	12	1	36	10
**Age (yrs.)**	43 ± 11	62 ±8	55 ±10	56 ±10
**Body mass index (kg/m^2^)**	30.6 ± 4.5	28.6 ± 4.4	30.5 ± 4.7	29.3 ± 5.9
**Weight (kg)**	93 ± 17	78 ± 11	89 ± 17	85 ± 23
**Height (cm)**	174 ± 10	165 ± 6	171 ± 9	169 ± 10
**Waist circumference (cm)**	100 ± 17	98 ± 12	103 ± 14	105 ± 21
**Waist-to-height ratio (cm/cm)**	0.57 ± 0.08	0.60 ± 0.09	0.60 ± 0.08	0.62 ± 0.11
**aMMP-8 levels**				
aMMP-8 ≥ 20 ng/mL	2	2	31	17
aMMP-8 < 20 ng/mL	29	12	50	6
**Stage of periodontitis status**				
No evidence of periodontitis	31	0	0	0
Stage I	0	14	0	0
Stage Il	0	0	81	0
Stage III	0	0	0	23
**Grade of periodontitis status**				
No evidence of progression	31	0	0	0
Grade A	0	7	7	0
Grade B	0	7	70	13
Grade C	0	0	4	10
**CAL (mm)**	2.4 ± 0.5	2.3 ± 0.5	3.4 ± 0.8	4.8 ± 1.2
**PPD (mm)**	2.2 ± 0.3	2.2 ± 0.4	3.0 ± 0.7	3.9 ± 0.9
**Number of teeth present (No.)**	27 ± 2	25 ± 2	24 ± 3	22 ± 4
**VPI (%)**	43 ± 22	29 ± 20	48 ± 27	63 ± 28
**BOP (%)**	42 ± 25	37 ± 18	56 ± 23	62 ± 23

**Table 2:** Coefficient output data of multiple logistic regression modeling of the two functions created.

Model and Variables	B	S.E.	Wald	df	Sig	Exp(B)
**Function separating healthy and stage I patients from stage II and III patients**						
aMMP-8 test (cut-off: 20 ng/mL) or diabetic	1.997	0.598	11.150	1	<0.001	7.365
Age × waist-to-height ratio cut-off 26 yrs. × cm/cm	1.615	0.522	9.566	1	0.002	5.029
Patient’s VPI% < 50% and annual dental visit	1.653	0.483	11.717	1	<0.001	5.221
No missing teeth and tobacco non-smoker	1.180	0.526	5.045	1	0.025	3.256
Constant	−2.649	0.675	15.403	1	<0.001	0.071
**Function separating stage II and III patients**						
aMMP-8 test (cut-off: 20 ng/mL) or >6 teeth missing	1.719	0.624	7.592	1	0.006	5.581
Patient’s VPI% ≥ 70% or is a tobacco smoker or diabetic	1.988	0.617	10.362	1	0.001	7.297
Constant	−3.706	0.755	24.107	1	<0.001	0.025

## Data Availability

The data presented in this study are available on request from the corresponding author due to privacy and legal reasons.
